# A case report of midodrine to treat protein-losing enteropathy for heart transplant candidacy

**DOI:** 10.1093/ehjcr/ytaf544

**Published:** 2025-10-23

**Authors:** Sean M Conte, Daniel Tardo, Stephanie Wiltshire, Kavitha Muthiah

**Affiliations:** Department of Cardiology, St Vincent’s Hospital Sydney, 390 Victoria Street, Darlinghurst, NSW 2010, Australia; University of New South Wales, School of Medicine, Wallace Wurth Building (C27), Cnr High St & Botany St, UNSW Sydney, Kensington, NSW 2033, Australia; University of Notre Dame Australia, School of Medicine, 160 Oxford St, Darlinghurst, NSW 2010, Australia; Department of Cardiology, St Vincent’s Hospital Sydney, 390 Victoria Street, Darlinghurst, NSW 2010, Australia; University of New South Wales, School of Medicine, Wallace Wurth Building (C27), Cnr High St & Botany St, UNSW Sydney, Kensington, NSW 2033, Australia; University of Notre Dame Australia, School of Medicine, 160 Oxford St, Darlinghurst, NSW 2010, Australia; Department of Cardiology, St Vincent’s Hospital Sydney, 390 Victoria Street, Darlinghurst, NSW 2010, Australia; University of New South Wales, School of Medicine, Wallace Wurth Building (C27), Cnr High St & Botany St, UNSW Sydney, Kensington, NSW 2033, Australia; Department of Cardiology, St Vincent’s Hospital Sydney, 390 Victoria Street, Darlinghurst, NSW 2010, Australia; University of New South Wales, School of Medicine, Wallace Wurth Building (C27), Cnr High St & Botany St, UNSW Sydney, Kensington, NSW 2033, Australia; Victor Chang Cardiac Research Institute, Lowry Packer Building, 405 Liverpool St, Darlinghurst, NSW 2010, Australia

**Keywords:** Congenital heart disease, Heart failure, Heart transplant, Protein-losing enteropathy, Fontan, Case report

## Abstract

**Background:**

Protein-losing enteropathy (PLE) is seen in up to 15% of patients with Fontan circulation and has important nutritional, immunologic, and haemodynamic consequences especially in the context of advanced heart failure. Midodrine, an alpha-1-receptor agonist, increases lymphatic tone, mitigating leak of lymph into the bowel, and has been shown to have clinical and biochemical benefit in patients with Fontan failure.

**Case summary:**

A young woman with Fontan circulation, end-stage heart failure, and PLE developed progressive hypoalbuminaemia, deconditioning, and frailty prohibitive to transplantation candidacy. Three months after commencing oral midodrine therapy, she experienced normalization of her serum albumin and improvements in her oedema, exercise tolerance, and frailty enabling candidacy and eventual successful heart transplantation.

**Discussion:**

The development of PLE in patients with Fontan circulations is an indication for cardiac transplantation, but there are often significant barriers due to the many complications. We performed a comprehensive Medline search, and this is the first case demonstrating the use of midodrine to bridge a frail patient with PLE and advanced Fontan heart failure to heart transplantation candidacy and eventual transplant.

Learning points

- To understand the pathophysiology and clinical implications of protein-losing enteropathy in patients with Fontan failure.
- To understand the potential role of midodrine and other therapies in mitigating the consequences of protein-losing enteropathy in patients with Fontan failure.

## Introduction

Protein-losing enteropathy (PLE) is seen in up to 15% of patients with Fontan circulation and has important nutritional, immunologic, and haemodynamic consequences especially in the context of advanced heart failure therapies such as transplantation. Midodrine, an alpha-1-receptor agonist, increases lymphatic tone, mitigating leak of lymph into the bowel. We present a frail patient with advanced Fontan failure and PLE treated with midodrine, enabling transplant candidacy.

## Summary figure

**Figure:**
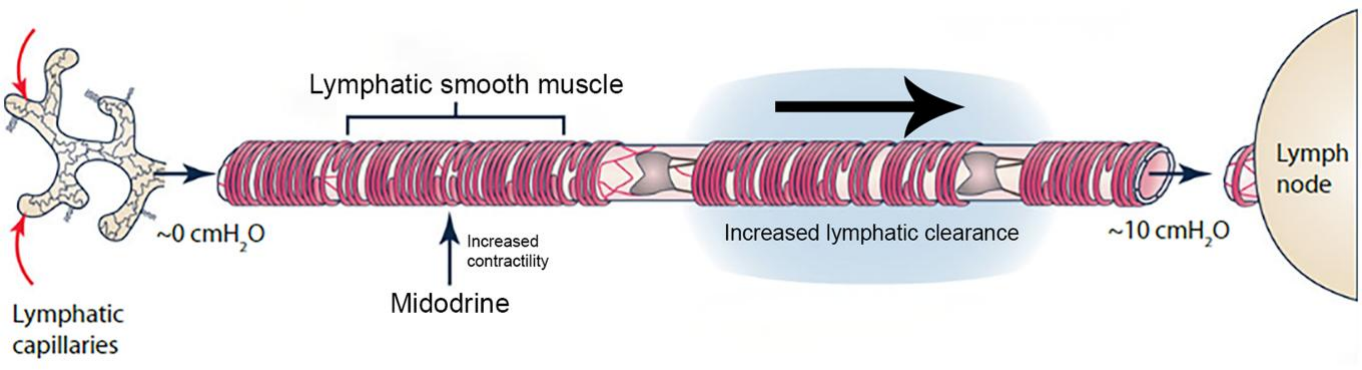
Mechanism of action of midodrine in reducing lymph leak into the bowel. (Adapted from Scallan *J Physiol* 2016, with permission).^1^

## Case presentation

A 20-year-old woman was referred to our advanced heart failure and heart transplantation service with New York Heart Association (NYHA) class III dyspnoea, recalcitrant lower limb oedema and ascites, and progressive congestive hepatopathy and cardiorenal syndrome. In the year before discharge, she had three hospitalizations for decompensated heart failure and fluid overload requiring intravenous diuresis. Her engagement with regular physiotherapy had waned over this period, and her exercise tolerance at the time of referral was 100–200 m with significant gait unsteadiness due to lower limb oedema. She was moderately frail by a validated pre-transplant screening test scoring four out of ten.^2^

Our patient had a history of hypoplastic left heart syndrome with prior Norwood procedure, bidirectional Glenn shunt, and aortic coarctation repair in infancy. She underwent Fontan completion with a fenestrated extra-cardiac conduit at the age of 6 years, and her last intervention was a balloon dilatation of her Damus–Kaye–Stansel anastomosis at age 8 years. She had a mild intellectual disability but was able to participate in cardiac rehabilitation up until the time of her referral to our centre.

The differential diagnosis for this patient included common causes of oedema and deconditioning in the heart failure population such as fluid overload, proteinuric renal failure, and hepatic synthetic dysfunction as well as PLE given her Fontan circulation.

Comprehensive investigations revealed low serum albumin of 16 g/l, negative 24 h urinary albumin, and positive faecal alpha-1 antitrypsin consistent with PLE. Right heart catheterization demonstrated mean right pulmonary artery pressure of 24 mmHg, mean left pulmonary artery pressure of 23 mmHg, Fontan pressure of 24 mmHg, and hepatic wedge pressure of 24 mmHg. Echocardiography revealed a severely dilated systemic ventricle with severe systolic dysfunction, a thickened previously repaired tricuspid valve with mild-moderate stenosis (mean gradient 4.8 mmHg) and mild regurgitation, and an estimated pulmonary artery systolic pressure of 86 mmHg.

Our patient’s frailty, malnutrition, and inability to engage with physiotherapy represented significant barriers to transplantation, the only viable therapy for her end-stage heart failure. She commenced midodrine 2.5 mg orally three times daily. To ensure tolerability, she was admitted to hospital electively for observation during the first 3 days of midodrine therapy. No acute hypertension or other complications occurred during this period. She was then followed up biweekly in our outpatient heart failure clinic and reported excellent compliance. Over the ensuing 3 months, her serum albumin rose with a concurrent improvement in her peripheral oedema. This resulted in greater exercise tolerance, better engagement with physiotherapy, and reduction in overall frailty. Due to ongoing NYHA class III symptoms, she was reassessed for transplant candidacy, deemed acceptable, and listed for heart transplantation. After 49 days on the waiting list, she received a heart transplant from a well-matched donor. Her serum albumin had risen from a nadir of 16 g/L prior to midodrine therapy to 33 g/L just prior to her heart transplant (standard reference range for serum albumin 33–48 g/L). At 6-month follow-up post-transplantation, our patient had a normal serum albumin level and a negative faecal alpha-1 antitrypsin indicating resolution of her PLE.^3^

## Discussion

**Figure 1:**
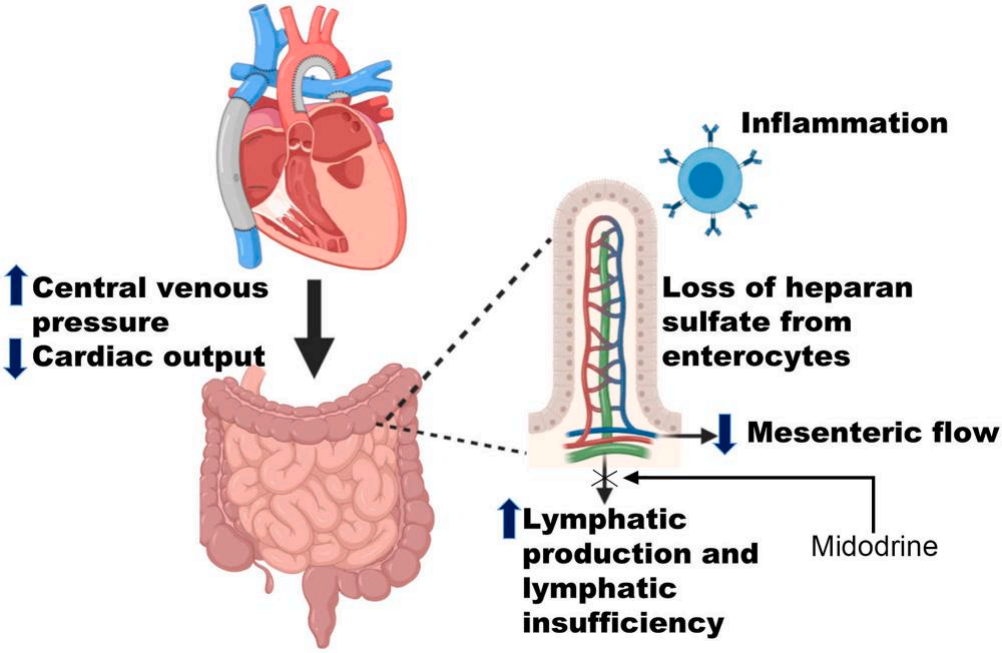
Mechanisms of protein-losing enteropathy in Fontan heart failure. (Adapted from Alsaied *Internat J Card Congen Heart Disease* 2002, with permission).^6^

Up to 15% of patients with Fontan circulations experience PLE which has important nutritional, immunologic, haemodynamic, and prognostic consequences.^4^ Indeed, the development of PLE in patients with Fontan circulations is an indication for cardiac transplantation, but there are often significant challenges to this pathway due to the complications of PLE.^5^ Characterized by excessive enteric protein losses and resultant hypoalbuminaemia, the pathophysiology of PLE is incompletely understood and ostensibly multifactorial: increased splanchnic oncotic pressure resulting from elevated central venous pressure as well as endothelial inflammation, loss of heparin sulfate from enterocytes, and lymphatic insufficiency due to mesenteric ischaemia and reduced cardiac output (*Figure 1*).^6^ In addition to diarrhoea, peripheral oedema, pleural and pericardial effusions, and ascites, excess gut protein loss causes hypogammaglobulinaemia, coagulopathy, and nutrient malabsorption all contributing to vulnerability and frailty in the Fontan cohort. Longer-term complications especially relevant in the paediatric population include failure to thrive, decreased bone mineral density, and attenuated growth. This combination of issues is strongly negatively prognostic: the 5-year mortality of Fontan patients who develop PLE is as high as 50%.^7^

Treatment of PLE in Fontan patients is multimodal and emphasizes optimizing nutrition via a low-fat, high-protein, high medium-chain triglyceride, low long-chain triglyceride diet, as well as management of underlying heart failure. Several medications have demonstrated biochemical improvement in small, case-based studies including octreotide, a somatostatin analog which reduces splanchnic blood flow and therefore lymphatic pressure^8^ and oral budesonide, a corticosteroid with low bioavailability that attenuates enteral inflammation, and therefore lymphatic losses.^9^ Subcutaneous unfractionated heparin has been associated with improvement in PLE symptoms thought to be due to its stabilizing effect on the gastrointestinal capillary endothelium.^10^ The development of PLE in patients with Fontan circulations is ultimately a poor prognostic marker and a signal that definitive therapy is imminently required.

Midodrine is an alpha-1-receptor agonist which increases lymphatic tone by increasing lymphatic smooth muscle contractility and enhancing the active pumping of lymph. In the setting of PLE where there is elevated lymphatic afterload and a higher hydrostatic gradient, both leading to reduced passive lymph flow, this improves lymphatic clearance, decreases stasis, and mitigates leak of lymph into the bowel.^1^ Midodrine has been used in Fontan patients with PLE and resulted in marked clinical and biochemical improvement in a recent case series.^11^ Indeed, midodrine may be helpful in patients with hypoalbuminaemia and chronic systemic venous hypertension and PLE due to other aetiologies such as right heart failure, cor pulmonale, and chronic renal failure. Although generally well tolerated in patients with heart failure, midodrine can cause significant hypertension which can place additional afterload strain on the failing ventricle. Here, we present a patient with advanced Fontan heart failure and PLE in whom midodrine therapy was used as a bridge to heart transplantation candidacy. After several months of therapy with midodrine, she achieved a degree of biochemical and clinical improvement that enabled listing and eventual heart transplantation.

We performed a comprehensive Medline search, and this is the first case demonstrating the use of midodrine to bridge a frail patient with PLE and advanced Fontan heart failure to heart transplantation candidacy and eventual transplant. The case series mentioned above outlines the use of midodrine in patients with Fontan heart failure; however, none of these patients went on to receive transplantation as they improved to the point of not requiring transplant evaluation or listing. In our case, resolution of hypoalbuminaemia, reduction in oedema, improvement in engagement with physiotherapy, and reduction in frailty associated with midodrine therapy enabled eligibility for heart transplantation listing as well as ongoing pre-habilitation prior to the transplant itself. Midodrine may be a useful tool for frail, hypoalbuminaemic patients with PLE and advanced Fontan heart failure who may be borderline or ineligible for heart transplantation. Larger controlled studies are needed to strengthen evidence for midodrine therapy in this population. Ongoing work elucidating mechanisms of benefits from midodrine therapy will be beneficial to establish the role of midodrine in the comprehensive management of such patients.

## Data Availability

The data underlying this article cannot be shared publicly as it is contained within our patient’s medical record; however, annonymized laboratory data can be shared on reasonable request to the corresponding author.

## Lead author biography

Sean Conte, BA, MBBS, FRACP completed his cardiology training at St Vincent’s Hospital Sydney in 2024. He completed his medical education at University of Sydney and internal medicine at St Vincent’s Hospital Sydney. His interests include advanced heart failure and transplantation, interventional cardiology, and medical education.

## Notes

**Consent:** The authors confirm that written consent for submission and publication of this case has been obtained from the patient in line with COPE guidance.

**Funding:** None declared.
